# A Cylindrical Triode Ultrahigh Vacuum Ionization Gauge with a Carbon Nanotube Cathode

**DOI:** 10.3390/nano11071636

**Published:** 2021-06-22

**Authors:** Jian Zhang, Jianping Wei, Detian Li, Huzhong Zhang, Yongjun Wang, Xiaobing Zhang

**Affiliations:** 1 School of Electronic Science and Engineering, Southeast University, Nanjing 210096, China; contact_zhangjian@163.com; 2 Science and Technology on Vacuum Technology and Physics Laboratory, Lanzhou Institute of Physics, Lanzhou 730000, China; m18856325392@163.com (J.W.); lidetian@hotmail.com (D.L.); janehuge@126.com (H.Z.)

**Keywords:** ionization gauge, CNT, field emission, pressure measurement, lower limit

## Abstract

In this study, a cylindrical triode ultrahigh vacuum ionization gauge with a screen-printed carbon nanotube (CNT) electron source was developed, and its metrological performance in different gases was systematically investigated using an ultrahigh vacuum system. The resulting ionization gauge with a CNT cathode responded linearly to nitrogen, argon, and air pressures in the range from ~4.0 ± 1.0 × 10^−7^ to 6 × 10^−4^ Pa, which is the first reported CNT emitter-based ionization gauge whose lower limit of pressure measurement is lower than its hot cathode counterpart. In addition, the sensitivities of this novel gauge were ~0.05 Pa^−1^ for nitrogen, ~0.06 Pa^−1^ for argon, and ~0.04 Pa^−1^ for air, respectively. The trend of sensitivity with anode voltage, obtained by the experimental method, was roughly consistent with that gained through theoretical simulation. The advantages of the present sensor (including low power consumption for electron emissions, invisible to infrared light radiation and thermal radiation, high stability, etc.) mean that it has potential applications in space exploration.

## 1. Introduction

In recent years, ultrahigh and extremely high vacuum pressure measurements have become widely demanded in many science and engineering fields [1,2,3], including space exploration, surface science, particle accelerator, storage ring, etc. To date, hot cathode and cold cathode ionization gauges are the only practically available electronic vacuum sensors in this pressure region [4,5]. However, conventional ionization gauges have long-standing intractable problems when measuring extremely low pressure, i.e., X-ray effects, electron-stimulated desorption effects, and outgassing effects [1,6,7]. Fortunately, recent studies [8,9,10,11,12,13,14,15,16,17,18,19] have shown that ionization gauges with carbon nanotube (CNT) cathodes have unique advantages, such as low power consumption for electron emissions, fast responses, free from visible to infrared light radiation and thermal radiation, etc., in extremely low pressure measurements, which are largely due to the application of the novel CNT field emission cathode. For instance, Murakami et al. [9] reported the application of a CNT field emission cathode in a Bayard–Alpert gauge (BAG) for the first time in 2001, and the lower limit of pressure measurement by this novel sensor was approximately 1 × 10^−4^ Pa. Dong et al. [8] reported on the design and investigation of a commercial extractor gauge (Leybold, IE 514) with a CNT cathode, which showed excellent measurement linearity from 10^−10^ to 10^−6^ Torr in nitrogen. Sheng et al. [10] described a high-sensitivity saddle field gauge with a CNT cathode, and a linear pressure response was achieved from 10^−5^ to 10^−2^ Pa. In theory, the CNT cathode ionization gauges have several advantages; in reality, however, none of the reported ionization gauges with CNT cathodes have a lower limit of pressure measurement than that of corresponding hot cathodes with the same structural types. For example, the lower limit of a commercial extractor gauge (Leybold, IE 514) is as low as ~10^−10^ Pa, but the lower limit achieved by the extractor gauge with a CNT cathode in Ref. [8] was only 4 × 10^−8^ Pa. Likewise, in Ref. [18], the lower limit achieved by BAG with a CNT cathode was approximately 1 × 10^−4^ Pa; the value for the hot BAG cathode was about 1 × 10^−9^ Pa. In this sense, the advantages of the application of such novel CNT field emission cathodes in extremely low pressure measurements have not yet been fully demonstrated.

In this work, a high-quality CNT electron source was produced by a screen-printing method, and a cylindrical triode ultrahigh vacuum ionization gauge with a screen-printed CNT cathode was further developed by replacing the original hot filament with the resulting CNT electron source in order to demonstrate the advantage of an ionization gauge equipped with a CNT cathode in suppressing X-ray effects; the sensing characteristics of this novel gauge in nitrogen, argon, and air were evaluated systematically. The effects of anode voltage on gauge sensitivity were also studied by using the theoretical simulation method. Here, a lower limit of ~3.0 × 10^−7^ Pa was achieved in nitrogen using the present sensor under optimized conditions, which is the first reported CNT emitter-based ionization gauge whose lower limit of pressure measurement is lower than its hot cathode counterpart.

## 2. Materials and Methods

### 2.1. CNT Electron Source and Its Field Emission Characteristics

In this study, a novel field emission cathode was fabricated by screen-printing CNT paste on one end of a 5 mm diameter stainless steel rod. Here, the CNT paste, roughly 100 ± 20 μm in thickness, was composed of multi-walled CNTs of 10~15 nm in diameter, with inorganic fillers of alloy, and organic powder of ethyl cellulose and terpineol; the latter was used as a solvent. The CNT paste was formulated by using only ball-milling apparatus. The inner and outer diameters of the utilized CNT were ~7.0 and ~12.8 nm, respectively, and the number of walls was ~8. More detailed descriptions about the CNT cathode preparation processes are presented in Refs. [20,21]. The microstructure and morphology of the CNT cathodes were characterized by a LabRAM HR800 micro-Raman spectrometer (HORIBA Jobin Yvon, Edison, NJ, USA) operating with a 532 nm Ar^+^ laser as the excitation and a field emission scanning electron microscope (FE-SEM, Quanta FEI 200, Eindhoven, The Netherlands). The Raman spectra were recorded from ~75 to 3000 cm^−^^1^, and the scanning time for each sample was 30 s. Finally, the Raman spectra were fitted based on two Gaussian shape curves with a curve-fitting software to identify the peak positions and calculate the intensity ratio of D and G peaks.

The field emission electron source was constructed with a gate electrode and a CNT cathode, and they were 200 μm away from each other. The gate electrode, used to extract and accelerate the electron from the CNT emitters, was made of molybdenum sheet (15 mm in diameter, 50 μm in thickness), and the center of the molybdenum sheet, ~8 mm in diameter, was a mesh structure with ~80% physical transparency. The gate and the CNT cathode were electrically separated by insulating ceramics. The field emission properties of the resulting CNT electron source, including I-V characteristics and stability, were investigated in an ionization gauge, which is described in the next section in detail. In order to realize a reliable pressure measurement, the CNT electron source was firstly fired at 25 °C for 2 h in a muffle furnace in air in order to evaporate the organic residual slurry to reduce the outgassing from paste during field emission processes; then, it was conditioned in a vacuum of ~10^−8^ Pa at a current density of ~1 mA/cm^2^ for 10 h before it was used in the ionization gauge.

### 2.2. Cylindrical Triode Ionization Gauge with a CNT Electron Source

#### 2.2.1. Experimental Study

**Figure 1:**
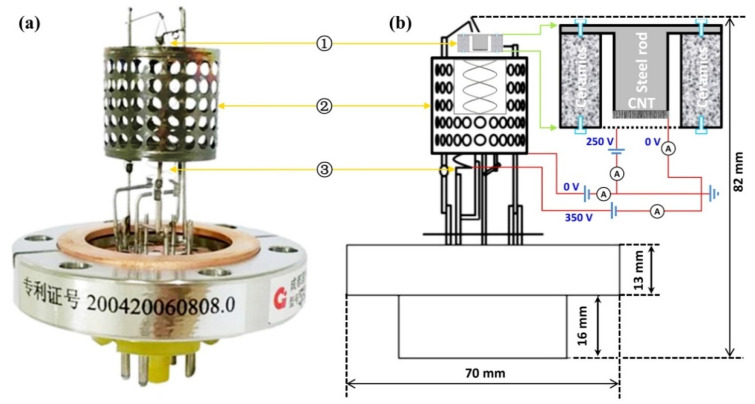
The original cylindrical triode ionization gauge (**a**), the schematic diagram of the modified ionization gauge with a CNT cathode (**b**), and the inset in (**b**) is the schematic illustration of the screen-printed CNT electron source used in this work. Here, ① hot filament/CNT electron source; ② cylindrical collector; and ③ helix-shaped anode. In addition, the electrode voltages used in the pressure sensing experiment are also given (blue numbers). The Chinese characters in (**a**) are patent number 200420060808.0.

In this work, a commercialized hot cathode ionization gauge with a triode structure (ZJ-27, Guoguang Electric Co. Ltd., Chengdu, China) was modified by replacing the hot filament with a screen-printed CNT electron source. The modified ionization gauge was mainly composed of a screen-printed CNT electron source, a cylindrical collector, and a helix-shaped anode grid. The gate electrode was 2 mm away from the anode apex. The helix-shaped anode, ~8 mm in diameter, and the cylindrical collector, ~25 mm in diameter, were coaxial, and situated ~8.5 mm away from each other. The measurement range of the original hot cathode ionization gauge (ZJ-27) was from 1.0 × 10^−5^ to 1.0 × 10^1^ Pa, with a sensitivity of 0.15 Pa^−1^ [22]. The gauge performances were evaluated in an ultrahigh vacuum system with an ultimate pressure of 1 × 10^−8^ Pa, which was monitored by a calibrated extractor gauge (Leybold, IE 514). The experimental gases (N_2_, Ar and air) were introduced into the system via a needle inlet valve to obtain the desired pressures (given by the nitrogen equivalent value). The voltages applied to the gate and the helix-shaped anode were provided by two Keithley 2290-5 source meters, and the cylindrical collector as well as the CNT cathode was grounded. The small ion collector current, *I_collector_*, was measured with a picoam-meter (Keithley 6487), and the CNT cathode current, *I_cathode_*, and the helix-shaped anode current, *I_anode_*, were detected using two high-precision digital multi-meters (FLUKE,17B). Finally, in order to determine the linear pressure measurement range of the present ionization gauge with a CNT cathode, the background signal obtained under the ultimate pressure condition was processed with the same procedure as described in Refs. [14,23]. The original cylindrical triode ionization gauge and the schematic diagram of the modified ionization gauge with a CNT electron source used in this work are illustrated in Figure 1.

#### 2.2.2. Theoretical Simulation

In order to investigate the effect of anode voltage on gauge sensitivity, we built the gauge model using SolidWorks 3D Modeling Software according to the structure parameters of the real ionization gauge with a CNT cathode, as shown in Figure 1b, and Ions Optic Software Simion 8.1 was used to determine the effective electron path length and the mean electron energy along its trajectory as a function of anode voltage; the ionization cross-section for the studied gas was further calculated by using an analytical formula obtained from the so-called binary encounter Bethe (BEB) model [24,25]. In this model, the ionization cross-section is calculated by each molecular orbital and then summed over all orbitals, which is finally given as: $\sigma_{\mathrm{BEB}}=\frac{K}{t+u+1}\left[\frac{\ln t}{2}\left(1-\frac{1}{t^{2}}\right)+1-\frac{1}{t}-\frac{\ln t}{t+1}\right]$; here, t = T/B, u = U/B, $K=4{\pi a}_{0}^{2}{\mathrm{NR}}^{2}/B^{2}$, $a_{0}=0.5292Å$, R = 13.61 eV. T is the incident electron energy (eV), B and U are the binding energy (eV) and kinetic energy (eV) of an electron on a given molecular orbital, respectively, and N is the number of electrons on the orbital [24]. By using the above parameters, the gauge sensitivity was calculated as follows:

*S* = (*L*·σ)/k*T* (1)

Here, *S* is the gauge sensitivity, *L* is the effective electron path length, which is defined as the electron trajectory length in the effective ionization space, k is the Boltzmann constant, *T* is the absolute temperature of molecules in the ionization region, and σ is the electron-impact ionization cross-section of gas molecules [26].

**Figure 2:**
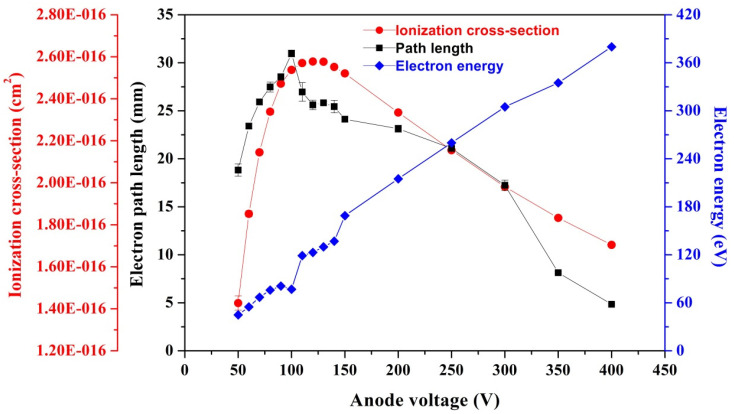
The stimulated electron-impact ionization cross-section, the mean electron path length, and the mean electron energy along electron trajectories as a function of anode voltage. The error bars are the standard deviation obtained by three calculations for the corresponding physical values.

In the simulation procedure, in order to improve the calculation accuracy and the reliability of the simulation results, a three-dimensional structural model was adopted. The grids of the calculation model for the studied ionization gauge were all hexahedral, and the total grid number was about 10^8^. The more detailed calculation methods were adequately described in our previous papers [12,27]. The gate voltage, the collector voltage, and the CNT cathode voltage were assumed to be 300, 0, and 0 V, respectively, and the studied anode voltage increased from 50 to 400 V. A total of 600 electrons with initial energies of 0.1 eV were randomly arranged on the CNT tips. The effect of anode voltage on the ionization cross-section, the effective electron path length, and the mean electron energy along electron trajectories were stimulated, and the corresponding results are presented in Figure 2. Here, the ionization cross-section and the effective electron path length will be used to calculate the gauge sensitivity.

## 3. Results and Discussion

### 3.1. Characterization of the Screen-Printed CNT Cathode

**Figure 3:**
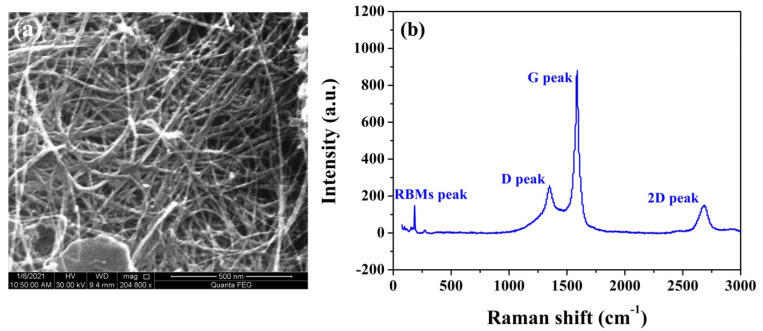
Microstructure characterization of the resulting CNT cathode. (**a**) FE–SEM image, and (**b**) Raman analysis.

The morphology and crystallinity of the CNT cathode were characterized by FE-SEM and Raman techniques, respectively, and the corresponding results are presented in Figure 3. The present CNT cathode principally consisted of highly tangled, randomly oriented, dense CNTs together with a certain number of impurities, as shown in Figure 3a. It is also obvious that numerous CNT bodies protruded from the binder matrix, and the defects on CNT bodies in addition to the CNT tips could become the active electron emission sites during field emission processes [28,29]. In addition, small amounts of Bi blocks could be found on the cathode surface, which was used to enhance the connection between the CNTs and the substrate [21]. In order to gain more insights into the structural characteristics of the CNT cathode, we performed Raman analysis in addition to FE-SEM for the as-received cathode. Figure 3b shows the typical Raman spectrum of the present CNT cathode. There are two strong peaks and two weak peaks in the wavenumber range of 75 to 3000 cm^−1^. The weak peaks, present at around 100 to 300 cm^−1^, result from scattering by the radial breathing modes (RBMs), namely, coherent vibration in phases of the carbon atoms in the radial direction. The appearance of RBM peaks in the Raman spectrum indicated that there was the definite existence of few-walled CNTs in the cathode [13]. The peak located at around 2688 cm^−1^ was assigned to the 2D peak, which originated from a double resonance process involving two phonons of opposite wave vectors. The strong peaks at ~1348.0 cm^−1^ and ~1585.6 cm^−1^ are the so-called D and G peaks, respectively. The D peak is related to the breathing modes of six-fold carbon rings in carbonaceous materials, and the G peak is generated by the stretching mode of all pairs of sp^2^ atoms in both rings and chains of graphitic materials [30,31]. The intensity ratio of the G peak to D peak, *I*_G_/*I*_D_, is commonly used to estimate the structural perfection of CNTs, which was as high as 3.2 in this case, indicating that the used CNTs had a high crystalline perfection [13,32]. It is reasonable to believe that the good crystallization of the present CNTs endows the cathode with exceptional field emission performances [32,33].

**Figure 4:**
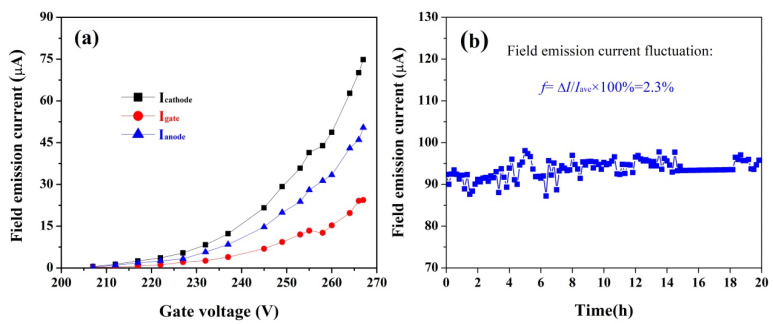
The field electron emission characteristics of the present CNT electron source. (**a**) I-V curve, and (**b**) stability.

The field electron emission properties of the integrated CNT electron source were evaluated in the modified cylindrical triode ionization gauge prior to vacuum pressure sensing application. In this process, the CNT cathode and the helix-shaped anode voltages were set to be 0 and 350 V, respectively, and the gate voltage gradually increased from 207 to 267 V; the corresponding results are given in Figure 4. It is observable from Figure 4a that the present CNT electron source exhibited typical field emission characteristics, because the emission current exponentially increased with increasing the gate voltage [34]. The maximal field emission current achieved at a gate voltage of 267 V is only 80 μA, because the gate voltage does not increase further in order to prevent the CNT from being degraded. The electron transmittance over the gate mesh is about 70% and is not strongly dependent on the gate voltage in the range from 207 to 267 V. In addition, in this case, the anode current reached up to 20 μA when the gate voltage was 250 V.

The field emission stability of a CNT electron source is of practical importance in vacuum electronic device applications. The short-term emission stability of the present CNT electron source was estimated by applying a constant gate voltage of 270 V for a period of 20 h. The initial emission current was set to be ~90 μA, which is a more stringent value than the one used in the next pressure sensing experiment. It is observable that, as demonstrated in Figure 4b, the emission current was almost constant over 20 h of continuous operation only with a minor emission fluctuation of 2.3% (the emission current fluctuation is defined as: *f* = △*I*/*I*_ave_ × 100%. Here, *f* is the emission current fluctuation, *I*_ave_ is the averaged field emission current during the whole test period, and △*I* is the standard deviation [32], indicating an excellent field emission stability. In fact, the outgassing from screen-printed CNT electron source is one of the main reasons of emission instability for such electron sources. In this work, the field emission stability of the CNT electron source was estimated after a series of experimental treatments, including high-temperature (250 °C) treatment, aging treatment, and an I-V test. Consequently, the heat induced during these treatment processes can accelerate the evaporation of residual organic slurry, and thus a stable emission characteristic of the present CNT electron source is obtained. It is universally recognized that the electron emission stability of a CNT electron source is one of the key factors for determining its potential application in devices; the good emission stability of an integrated CNT electron source is definitely favorable for the development of high-performance electric vacuum devices.

### 3.2. Metrology Behaviors of the Novel Ionization Gauge

For ionization gaugse, a low anode current (i.e., 20 μA) would greatly reduce the adverse impact of X-ray effects [12,18,35] in extremely low pressure measurements, even if it would cause difficulties due to weak collector current detection [7,8]. Thus, the metrological behaviors of the modified ionization gauge with a CNT electron source were investigated under a low anode current together with constant electrode voltages, as demonstrated in Figure 1b. In this situation, the anode current was only about 20 μA, which is high enough for extremely low pressure measurement, according to our previous experience [11,14,17].

**Figure 5:**
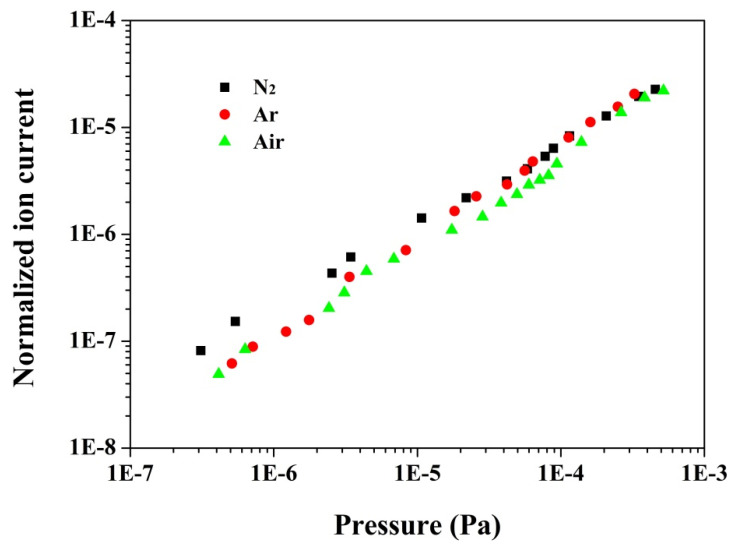
The normalized ion current of the modified ionization gauge with a CNT electron source as a function of testing pressure in different gases.

The sensing principle of an ionization gauge is described as follows: *I_ion_/(p × I_anode_) = S*, where *I_ion_* and *I_anode_* are the collector and the anode currents, respectively, *p* is the pressure, and S is the gauge sensitivity [5]. The pressure-sensing characteristics of the modified ionization gauge with a CNT electron source were investigated, and the corresponding results are presented in Figure 5. The normalized ion currents (*I_ion_*/*I_anode_*) for three testing gases (i.e., nitrogen, argon and air) are linear relative to the pressure in the range from ~4.0 ± 1.0 × 10^−7^ to ~6.0 × 10^−4^ Pa, indicating good characteristics for the pressure sensor. Here, it is worth noting that the lower limit of the studied ionization gauge is two orders of magnitude lower than its hot cathode counterpart [22]. To the best of our knowledge, this is the first reported CNT emitter-based ionization gauge whose lower limit of pressure measurement is lower than its hot cathode counterpart [5,36]. It is well-known that in cylindrical triode ionization gauges, X-ray effects are one of the most dominant factors to restrain the extension of the lower limit of pressure measurements of such a gauge [1,6]. This is mainly due to the fact that the X-rays mainly produced by electron-impact on the anode generate a photoelectron current from the ion collector, and thus produce a pressure-independent background current. This background current, in reality, is comparable to or even larger than the ion current originating from ionizing gas molecules in extremely high even ultrahigh vacuum pressure regions; consequently, a nonlinear characteristic in normalized ion current–pressure plots is generally observed in hot cathode ionization, as widely reported in previous studies [18,37,38]. In order to suppress the adverse impact of X-ray effects in very low pressure measurements, some measures (i.e., reducing the collector area, locating the collector out of the line of sight of the gauge grid and the filament, adding a suppresser electrode, and utilizing a current modulation technique) were employed to extend the lower limit of pressure measurements of the ionization gauge [6,35,39]; however, to date, the results have not been satisfactory. In the present work, a field emission CNT electron source was used, which has several advantages in extending the lower limit of pressure measurements of the modified ionization gauge. On the one hand, the novel CNT electron source successfully avoids any radiation (thermal, light and ultraviolet) effects which exist in hot cathodes, and thus the photoelectron current emitting from the collector induced by strong ultraviolet radiation does not exist in the present gauge, which is conducive to extending the lower limit of pressure measurements of the present gauge [40,41]. On the other hand, the present ionization gauge had a very low power consumption, of about 9.5 mW, compared with ~3.45 W of the cylindrical triode ionization gauge with a hot cathode [22]; as a result, outgassing of the gauge materials induced by thermal radiation or thermal conductivity would be extremely low, which is favorable for achieving extremely low pressure measurements [7,36,42]. Additionally, the X-ray photocurrent was proportional to the anode current [43], which was as high as 1 mA in a cylindrical triode ionization gauge with a hot cathode [22]. In this work, as mentioned above, the anode current was about 20 μA, only one-fiftieth times lower than that for the cylindrical triode hot cathode ionization gauge, which is conducive to greatly reducing the X-ray photocurrent, and is thus beneficial for obtaining lower limits of pressure measurements for the present gauge. The sensitivity values of the ionization gauge, derived from the slopes of the linear regions in Figure 5, were ~0.05 Pa^−1^ for nitrogen, ~0.06 Pa^−1^ for argon, and ~0.04 Pa^−1^ for air, which are even slightly higher than that of the most prevalent CNT cathode extractor gauge [8,11,12,17] and BAG [16], and are acceptable values for extremely low pressure measurements [7]. The detailed variation of sensitivity of this ionization gauge will be further discussed below.

**Figure 6:**
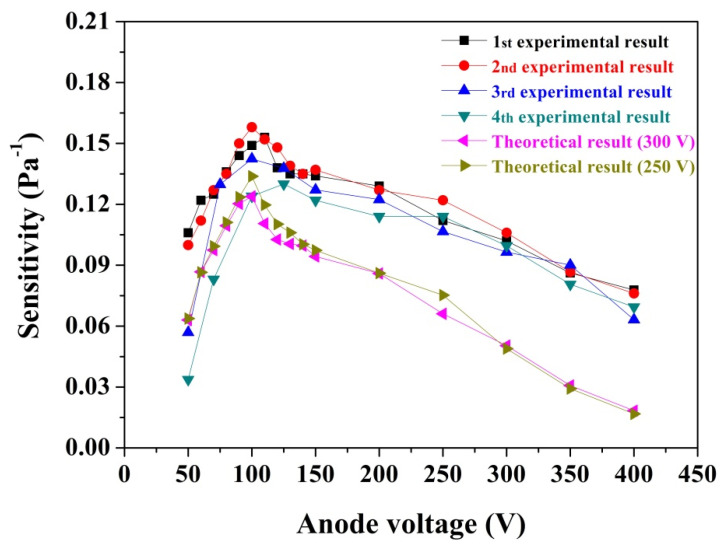
The experimental and simulated sensitivities of the studied ionization gauge with a CNT cathode as a function of anode voltage.

The gauge sensitivity is a key parameter for pressure sensing, which is related to several factors, including the gauge physical structure, the electrode voltage, and the gas species [5,10,14]. In general, a high sensitivity is necessary for an ionization gauge with a low anode current to monitor the extremely low pressure [44]. In this work, the effect of anode voltage on gauge sensitivity was investigated by both experimental and theoretical methods. In the experimental study, the variation of gauge sensitivity as a function of anode voltage was investigated under a vacuum pressure of ~6.3 × 10^−5^ Pa, the measurement was repeated four times to confirm the repeatability of the experimental result, and the corresponding results are presented in Figure 6. Here, in order to ensure that the anode current was consistent with the value in Figure 5, the gate voltage is set to be 300 V in this case. It is apparent that under a constant gate voltage, the gauge sensitivity increases as the anode voltage increases from ~50 to ~100 V, and then gradually decreases as the anode voltage increases further. In a hot cathode ionization gauge, the highest sensitivity is usually obtained with an anode voltage of ~100 to 150 V (the corresponding electron energy is in the range from 100 to 150 eV) for most pervasive gas molecules [13]. However, in the ionization gauge with a CNT cathode, the situation is more complicated because the emitting electron generated from CNT emitters is not only accelerated by the gate voltage, but is also impacted by the anode voltage. In our case, the highest sensitivity, ~0.15 Pa^−1^, was achieved at an anode voltage of ~100 V, which is comparable to the sensitivity of ZJ-27 commercial hot cathode gauges (~0.15 Pa^−1^) [22]. Here, it should be pointed out that the sensitivity obtained from Figure 5 is lower than that of the value given in Figure 6, which is mainly attributed to the fact that the sensitivity in Figure 5 is derived from the slope of normalized ion current–pressure plot. This is a relatively averaged value corresponding to the full pressure region from ~4.0 × 10^−7^ to ~6.0 × 10^−4^ Pa; however, the sensitivity in Figure 6 is obtained by single point calculation, namely, it is achieved at a pressure of ~6.3 × 10^−5^ Pa. It can be seen from Figure 5 that the slope in the high-pressure region (*p* > 3 × 10^−5^ Pa) is clearly higher than that of the low-pressure region (*p* < 3 × 10^−5^ Pa), and hence the sensitivity achieved from single point calculation at a pressure of ~6.31 × 10^−5^ Pa is higher relative to the averaged value obtained from the full pressure region. At constant gate voltages (250 and 300 V), the trend of sensitivity with anode voltage, obtained by the experimental method, is consistent with the one gained by theoretical simulation, as demonstrated in Figure 6. It is generally believed that the gauge sensitivity is determined by the ionization cross-section of gas molecules as well as the effective electron path length, i.e., the gauge sensitivity is the integral over the path of the electrons of the probability of ionization of a gas molecule by an electron [45]. Thus, it can explain the variation of the experimental sensitivity with anode voltage. As is clearly shown in Figure 2, both the electron-impact ionization cross-section and the effective electron path length reach their maximum values at an anode voltage of ~100 V. Consequently, the highest sensitivity is naturally obtained according to Equation (1). In addition, it should be noted that under some anode voltages (e.g., 250 V), the sensitivity derived from experimental testing was higher than the one obtained by theoretical simulation, which is mainly because in the theoretical simulation, only the contribution of gas phase ions was considered. However, in the experiment, the contributions from ions generated by electron-stimulated desorption and weak X-ray effects were also included, except for gas phase ions; hence a higher sensitivity was achieved. It is reasonable that the highest sensitivity should be used in pressure sensing in our work; however, when the anode voltage was 100 V, the anode current was very low, ~5.4 μA. As a result, the collector current for the pressure measurement of 3.0 × 10^−7^ Pa was extremely low, which poses a huge challenge for detecting the weak current signal using the picoam-meter, and is also the reason for the unstable sensitivity in the low anode voltage region, especially at 50 V, as shown in Figure 6. In order to keep the ion current signal intensity at a sufficient level, a compromise is needed between the sensitivity and the ion current; therefore, an anode voltage of 350 V was used in this study.

## 4. Conclusions

In summary, an ionization gauge with an integrated CNT electron source was developed in this work. The field emission performances of the integrated CNT electron source, as well as the metrological behaviors of this novel ionization gauge, were investigated. The electron source exhibited good field emission characteristics with excellent stability, and the lower limit of pressure measurement of the ionization gauge with a CNT cathode was as low as ~3.0 × 10^−7^ Pa in nitrogen; this is the first CNT emitter-based ionization gauge with a lower limit of pressure measurements than its hot cathode counterpart. The gauge sensitivity is anode voltage-dependent, and a highest value of ~0.15 Pa^−1^ was achieved at an anode voltage of ~100 V. In addition, the resulting ionization gauge with a CNT electron source had an extremely low power consumption of about 9.5 mW, which makes it advantageous over hot cathode ionization gauges in space exploration.

## Data Availability

Data are available from Y.W. upon reasonable request.
